# Cluster of human parechovirus infections as the predominant cause of sepsis in neonates and infants, Leicester, United Kingdom, 8 May to 2 August 2016

**DOI:** 10.2807/1560-7917.ES.2016.21.34.30326

**Published:** 2016-08-25

**Authors:** Julian W Tang, Christopher W Holmes, Fadwa A Elsanousi, Ayushi Patel, Fazila Adam, Rachel Speight, Savitha Shenoy, Daniel Bronnert, Gary Stiefel, Premkumar Sundaram, Suchandra Pande, Arani Sridhar, Venkatesh Kairamkonda, Srini Bandi

**Affiliations:** 1 Clinical Microbiology and Virology, University Hospitals of Leicester NHS Trust, Leicester, United Kingdom; 2 Infection, Immunity and Inflammation, University of Leicester, Leicester, United Kingdom; 3 Leicester Childrens Hospital, University Hospitals of Leicester NHS Trust, Leicester, United Kingdom

**Keywords:** parechovirus, neonates, infants, cerebrospinal fluid, sepsis, PCR

## Abstract

We report an unusually high number of cases (n = 26) of parechovirus infections in the cerebrospinal fluid (CSF) of neonates and infants admitted with sepsis in the United Kingdom during 8 May to 2 August 2016. Although such infections in neonates and infants are well-documented, parechovirus has not been routinely included in many in-house and commercial PCR assays for CSF testing. Clinicians should consider routine parechovirus testing in young children presenting with sepsis.

Parechoviruses usually causes self-limiting, mild gastroenteritis and respiratory infections, though more severe neurological and cardiovascular complications are possible. We report a sudden and unusual increase in the number of cases of human parechovirus (HPeV) infection in neonates and infants admitted to hospital with sepsis during May to August 2016, in Leicester, United Kingdom (UK). 

The aim of this report is to alert other teams in Europe and elsewhere, who may not test for HPeV routinely, either in respiratory, enteric or cerebrospinal fluid (CSF) samples, in neonates and infants admitted to hospital for respiratory illness, gastroenteritis or sepsis.

## Detection of a cluster of parechovirus infections

In this case series, human parechovirus PCR testing on CSF was a routine part of the septic workup for any neonate or infant admitted to hospital presenting with any combination of fever, lethargy or drowsiness, rash, poor-feeding, tachycardia and irritability.

During routine diagnostic testing of neonates and infants admitted with suspected sepsis, where CSF was taken and tested as part of the septic workup, we confirmed 26 cases (15 male, 11 female) of HPeV infection in neonates and infants aged between 8 and 197 days (median: 47). 

This is in contrast to previous years: in 2015, one case was diagnosed (in July), in 2014, 10 cases were diagnosed over a four-month period (between March and July), in the same hospital using the same assay and clinical testing algorithm. The unusual aspect of this cluster was the sudden appearance of multiple cases within this short three-month period (May to August 2016) (Figure).

**Figure:**
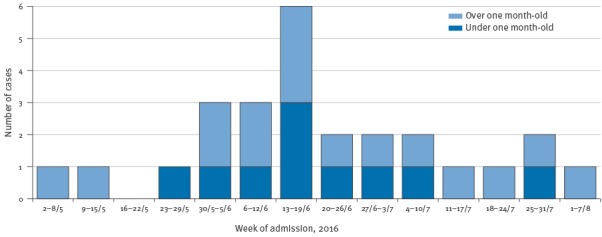
New cases of human parechovirus infection in neonates and infants admitted with sepsis, Leicester, United Kingdom, 8 May–2 August 2016 (n = 26)

The first case was reported on 8 May, the last on 2 August 2016.

These cases were diagnosed by testing of CSF samples using a combination of multiplex PCR assays. This included a commercial polymerase chain reaction (PCR) assay for the detection of enterovirus and HPeV (FTD EPA, Fast-track diagnostics Ltd, Sliema, Malta). Although this kit is marketed specifically for respiratory and stool specimens, we have internally validated it for CSF testing also, to take advantage of the EV and HPeV components. While adenovirus is also part of this kit, this target is not routinely screened for in standard CSF panels, so this component of the kit was not used. In all of these cases, all the other targets in our CSF test panel (herpes simplex virus (HSV) 1 and 2, varicella zoster virus (VZV) and enterovirus) were screened for using an in-house assay, and were negative. The in-house HSV-1, HSV-2 and VZV PCR assays were adapted from previously published protocols [1,2].

All 26 cases presented with very similar symptoms of generalised sepsis, including high fever (up to 40 °C), lethargy or drowsiness, poor feeding, tachycardia, grunting, mottled or petechial rash and irritability, with no other viral or bacterial agent found in systemic samples (i.e. by PCR testing or blood cultures).

In most of these cases (n = 24), the CSF glucose and protein levels were within normal limits, and all but one (one sample could not be tested as it was clotted) had a total white cell count of <10 (Table). Just under half of the patients (n = 11) had moderately elevated levels for liver function tests (alanine aminotransferase or total bilirubin) (Table). In addition, in two cases, their gamma-glutamyl transferase level was elevated (80 and 200 IU/L; norm: 0–35 IU/L) and in another two, their alkaline phosphatase level was raised (424 and 529 IU/L; norm: 60–245 IU/L).

**Table:** Age, duration of hospital stay and key laboratory parameters for 26^a^ cases of human parechovirus infection, Leicester, United Kingdom, 8 May–2 August 2016

Parameter	Median (range)
Age, in days	35 (8–197)
Duration of hospital stay, in days	4 (2–10)
C-reactive protein Norm: 0–10 mg/L	<5 (<5–40) mg/L
Total white cell count Norm: 6.0–17.5 × 10^9^/L	6.3 (2.6–17.4) × 10^9^/L
Lymphocytes Norm: 4.0–13.5 × 10^9^/L	1.93 (0.91–3.59) × 10^9^/L
Neutrophils Norm: 1.0–8.5 × 10^9^/L	2.91 (1.20–13.92) × 10^9^/L
Platelets Norm: 140–400 × 10^9^/L	327 (174–661) × 10^9^/L
Alanine transferase^b^ Norm: 5–100 IU/L	24 (9–359) IU/L
Total bilirubin^b^ Norm: 0–21 µmol/L	15 (4–217) µmol/L
Cerebrospinal fluid	
Glucose Norm: 2.5–4.4 mmol/L	3.1 (2.1–4.1) mmol/L
Protein Norm: 0.2–0.8 g/L	0.38 (0.20–1.56) g/L
Total white cell count^c^ Norm: 0 × 10^6^/L	1 (0–4) × 10^6^/L
Red blood cells^c^ Norm: 0 × 10^6^/L	3 (0–5,520) × 10^6^/L

CSF: cerebrospinal fluid; IU: international units.

^a^ Unless otherwise indicated.

^b^ For 21 of the 26 patients (not all patients were tested for all laboratory parameters, depending on the presentation of the patient).

^c^ For 25 of the 26 patients, as one sample was clotted.

While the initial presentation was of sufficient clinical concern to lead to hospital admission, in most cases, the disease settled without further complication. Most patients (n = 18) were discharged after two to four days. 

However, in one neonate, there was a more severe illness, with sepsis and encephalitis, requiring ionotropic support and ventilation. A tonic seizure occurred on day two of admission, and HPeV was detected in the CSF. Further testing detected HPeV in the stool, blood and a throat swab, confirming HPeV sepsis, and intravenous immunoglobulin was given. A follow-up electroencephalogram and magnetic resonance imaging of the brain both indicated encephalitis. The neonate was discharged after 10 days with no obvious neurological sequelae. 

## Background

Parechovirus is a non-enveloped, single-stranded RNA virus within the family *Picornaviridae*, which also includes rhinoviruses and enteroviruses. There are at least 16 different human HPeV types, of which HPeV type 3 is the most common cause of clinical disease in humans [3]. The spectrum of disease (mainly for HPeV 3) can range from self-limiting mild gastroenteritis and respiratory infections to more severe neurological complications (acute flaccid paralysis, encephalitis) and myocarditis [4]. 

Infections with HPeV in neonates and infants have been well-documented [5-10], but HPeV has only relatively recently been included as a target in our in-house and some commercial PCR assays used for testing CSF. This is most likely due to the growing recognition of HPeV as a common potential cause of sepsis and febrile seizures from various studies and outbreak investigations in recent years [11-16].

## Discussion

This increase in the number of HPeV infections associated with sepsis in neonates and infants is now being confirmed elsewhere in the UK, and viral sequencing analysis is currently in progress for samples from these 26 cases and others (David Allen, Public Health England, personal communications, July 2016).

Other recent reports of HPeV activity include an outbreak of HPeV infection in 55 neonates and infants (up to the age of three months) in Queensland, Australia, between September 2015 and February 2016 [17]. The presentation of these cases was very similar to that described for the 26 UK cases reported here (i.e. high temperature, diarrhoea, abnormally rapid breathing, severe irritability or appearing to be in pain, rashes or skin discolouration and jerking movements).

A recently published Norwegian study found HPeV in 9% (30/343) respiratory samples taken from 161 pre-school children and toddlers (aged 1–6.3 years), during a two-year study in which screening was carried out for 19 respiratory virus targets. This community-based study focused on relatively mild cases of respiratory infection that did not require medical attention outside of the study [18]. This is in contrast to our case series, in which HPeV was first tested and detected in CSF in neonates and infants who were considered ill enough to be admitted to hospital for investigation. In all but one of these cases, the illness was self-limiting and no further testing was required. In the one severely ill case described above, further HPeV testing was positive in stool, blood and a throat swab, confirming disseminated infection, which may have explained the severity of the illness. Transient viraemia may well have occurred in all these sample types in the other cases, but their self-limiting illness did not justify further sampling and testing for this.

Our routine PCR panel for testing respiratory samples is not validated for and therefore does not currently include HPeV, but in light of our findings reported here, we are now considering adding this. It is possible that HPeV may contribute to febrile seizures in young children that are often preceded by a non-specific febrile respiratory illness [19-21]. For this reason also, routinely including HPeV detection in our respiratory panel is being considered. 

From our experience with this ongoing case series of neonates and infants admitted for sepsis, we would recommend testing for HPeV in CSF, respiratory samples and/or stool samples, particularly for those patients presenting with unusually high fever and irritability, especially if no other pathogen can be identified. 

For other infants and other young children (older than 1 year), HPeV testing may be performed on stool samples if they present with gastroenteritis (abdominal pain, diarrhoea and vomiting); or on respiratory samples such as nasopharyngeal aspirates, if they present with respiratory symptoms (e.g. bronchiolitis and croup); or on CSF if they present with febrile seizures, and other aseptic meningitis symptoms (such as photophobia, lethargy, poor feeding and poor responsiveness). Again, all of these sample types can be tested for HPeV if generalised, systemic sepsis is suspected (which could result in a combination of all of these symptoms). 

Although HPeV testing in stool is the most useful to determine the duration of HPeV shedding for hospital infection control purposes, usually, these paediatric patients are discharged home as soon as they have recovered sufficiently, clinically, to minimise any onward transmission of HPeV to other patients on the ward.

In addition, as several cases exhibited tachycardia, a routine baseline electrocardiogram should also be recorded, as HPeV has been reported to cause cardiac problems [5,22]. Each of the 26 cases is currently under longer-term outpatient follow-up to check for any late central nervous or cardiovascular sequelae from this viral infection.

While no specific therapy is available, testing for HPeV as the cause of sepsis and/or encephalitis in these young children should be routine (along with testing for enteroviruses), even if the typical laboratory markers indicating sepsis may be relatively normal. This may reduce or prevent prolonged unnecessary empirical antibiotic treatment, thereby reducing the risk of antibiotic resistance arising, as well as optimising clinical care and the use of resources.
